# Dual-domain transformer-based learned primal-dual reconstruction for PET imaging

**DOI:** 10.3389/fnume.2026.1885873

**Published:** 2026-07-31

**Authors:** Anton Adelöw, Hamidreza Rashidykanan, Alessandro Guazzo, Massimiliano Colarieti-Tosti

**Affiliations:** 1 Department of Biomedical Engineering and Health Systems, KTH Royal Institute of Technology, Huddinge, Sweden; 2 Department of Radiology, University of Texas Southwestern, Dallas, TX, United States; 3 Department of Information Engineering, University of Padova, Padua, Italy; 4 Department of Clinical Science, Intervention and Technology, Karolinska Institutet, Stockholm, Sweden

**Keywords:** attention mechanism, cross-attention, deep learning, image reconstruction, learned primal-dual (LPD), MiniPET-3, positron emission tomography (PET), preclinical PET data

## Abstract

**Background and Objective:**

Positron Emission Tomography (PET) is widely used for assessing metabolic activity and diagnosing cancer. Due to the inherently high noise levels in PET data, advanced reconstruction algorithms are essential for accurate imaging. Convolutional Neural Networks (CNNs) within the Learned Primal-Dual (LPD) framework have shown good performance for PET reconstruction; however, the locality assumption imposed by CNNs can limit their ability to capture long-range contextual dependencies in sinogram data. Motivated by our previous work on transformer-based architecture for low-dose and low-count sinogram denoising, we comprehensively investigate the integration of attention mechanisms, both self-attention and cross-attention, within the LPD framework.

**Methods:**

We propose and evaluate three novel transformer-based LPD architectures: the Dual-Domain Stacked Transformer-based LPD, which employs sinusoidal patches to model long-range dependencies in the dual domain (sinogram); the Dual-Domain Restormer-based LPD, a hybrid design that combines CNNs for fine-grained local feature extraction with transformers for global information exchange; and the Dual-Domain UNet-based LPD incorporating Cross-Attention mechanism, which augments the U-Net LPD with cross-attention to capture complementary information across image and sinogram domains. To enable robust transfer learning, we further introduce a system-aligned synthetic data generation process that replicates the MiniPET-3 system, our in-house preclinical PET scanner dedicated to small-animal imaging, incorporating realistic acquisition geometry, resolution blurring, positron range effects, and Poisson noise.

**Results:**

Evaluations on synthetic data demonstrate that both the Stacked Transformer-based LPD and Dual-Domain Restormer-based LPD outperform the UNet-based LPD baseline in MSE and PSNR across all noise levels, with the Restormer additionally achieving higher SSIM at low noise. The UNet-based LPD incorporating a cross-attention mechanism also improves MSE and PSNR over the baseline, while exhibiting a slight reduction in SSIM, indicating a trade-off between pixel-wise accuracy and structural similarity. Trained exclusively on synthetic data, the proposed models generalize well to experimental measurements, producing high-quality reconstructions.

**Conclusions:**

Overall, this work highlights the promise of transformer-integrated LPD frameworks for enhancing PET reconstruction quality and robustness in low-count imaging settings.

## 1 Introduction

Positron Emission Tomography (PET) is a non-invasive molecular imaging modality widely used in oncology, neurology, and cardiology for studying metabolic and biochemical activity *in vivo*. In clinical PET, a fundamental tradeoff exists between image quality and acquisition constraints, as limitations on radiotracer dose and scan time often lead to low-count projection data. This low-count regime increases statistical noise, significantly degrading reconstruction quality and quantitative accuracy. Consequently, improving the reliability of PET image reconstruction from noisy measurements remains a central challenge [1, 2].

In recent years, deep learning has demonstrated strong potential in medical image reconstruction, particularly in CT and PET, significantly improving image quality [2–4]. Existing approaches can be broadly categorized based on how and where learning is applied within the reconstruction pipeline: (1) direct methods that map projection data to images in a fully data-driven manner [5–7], (2) projection-domain (denoising/filtering) methods that denoise sinograms prior to reconstruction, (3) post-processing methods that refine reconstructed images [8], (4) unrolled iterative methods that embed neural networks within iterative reconstruction algorithms, enabling the learning of components such as regularization or prior models [9, 10], (5) cross/dual-domain methods [11–22] that jointly exploit projection and image domains through cascaded [11–14, 22] or parallel [15–17, 21] arrangements, as well as hybrid combinations of both [20]. More recently, unrolled cross/dual-domain approaches have emerged, integrating dual-domain learning within an unrolled iterative framework to enable joint optimization across both domains [11, 12]. These categories highlight the evolution from purely data-driven approaches to more structured, unrolled cross/dual-domain iterative deep learning frameworks.

Among the aforementioned categories, cross/dual-domain and unrolled cross/dual-domain approaches have recently gained significant attention. Most prior works in these settings rely on CNN architectures, which often struggle to capture long-range dependencies and global context, potentially leading to reconstruction inaccuracies [23]. To address this limitation, recent studies have explored Transformer architectures, either as standalone models or in combination with CNNs and diffusion models.

Transformer-based CT and PET reconstruction methods can be broadly grouped into three categories: (1) pure Transformer-based methods, (2) hybrid Transformer–CNN methods, and (3) Transformer–diffusion models. In the first category, methods mainly focus on sinogram denoising. For example, Yang et al. [24] introduced the Sinogram Inner Structure Transformer (SIST) for low-dose CT (LDCT) reconstruction. In our prior work [22], we proposed a cross/dual-domain reconstruction network incorporating a Transformer-based sinogram denoising module designed to learn sinusoidal patterns. The second category includes hybrid methods combining CNNs and Transformers to exploit both local and global feature representations. For instance, [25] introduces a U-Net-based denoising network where CNN encoders extract fine image details while Transformer blocks in the bottleneck and decoder enhance structural representation via skip connections. Likewise, [26] integrates a residual U-Net–Transformer regularizer into an unrolled maximum a posteriori expectation maximization (MAPEM) framework for PET reconstruction. In [27], a residual CNN–Transformer strategy is used to predict sinogram residuals, while [20] combines Swin-Transformer and CNN modules for sinogram restoration. The third category includes methods integrating Transformers with diffusion models, combining strong representation learning with generative modeling capabilities [28, 29].

Although cross/dual-domain and unrolled cross/dual-domain approaches have recently gained significant attention, unrolled cross/dual-domain methods offer improved reconstruction quality by integrating domain-specific learning into an iterative, model-based framework that enforces data consistency and enables progressive joint optimization across both projection and image domains. The LPD algorithm [30] is a representative example of such unrolled cross/dual-domain frameworks, originally developed for CT reconstruction. LPD alternates between updates in the primal (image) domain and the dual (sinogram) domain, combining forward and backward projections with CNN-based feature extraction. Residual connections across iterations preserve information and stabilise training. Adaptations of LPD to PET [12] have also demonstrated that deep-learning-based unrolling can improve reconstruction quality over purely analytical or traditional iterative methods. However, CNN-based designs assumes that local feature correlations dominate and while this assumption holds for images, it is less appropriate for sinograms since pixels that are spatially close in an image will contribute to pixel values distributed over a large region in projection data space.

To address these limitations, we previously introduced Transformer-based sinusoidal curve learning for sinogram denoising [31]. Building upon this work and our prior study in [32], where self-attention was first incorporated into the LPD framework, as well as our earlier work in [21], where cross-attention was explored within a parallel cross-domain reconstruction framework using same-domain feature interactions, the present study extends this direction by systematically investigating both self-attention and cross-attention mechanisms in dual-domain unrolled iterative reconstruction. In contrast to [21], where cross-attention operates within a same domain, the proposed approach integrates cross-attention directly into the LPD architecture to enable interaction between the sinogram and image domains. Specifically, we propose three Transformer-based LPD architectures: Dual-Domain Stacked Transformer-based LPD, Dual-Domain Restormer-based LPD, and Dual-Domain U-Net-based LPD with a cross-attention mechanism.

In particular, we propose:

1. **Dual-Domain Stacked Transformer-based LPD:** a pure Transformer formulation that replaces local CNN operators with geometry-aware sinusoidal patch attention to capture global dependencies across dual domains.
2. **Dual-Domain Restormer-based LPD:** a hybrid design that combines convolutional feature extraction with Transformer-based global modeling to balance local and global representations.
3. **Dual-Domain U-Net-based LPD with Cross-Attention:** a model that explicitly models interactions between primal and dual domains through cross-attention, enabling direct exchange of complementary information across reconstruction spaces.

Together, these approaches represent three complementary perspectives on integrating attention mechanisms within the LPD framework. All proposed methods are evaluated on both 3D synthetically generated data and in-house preclinical PET data acquired from a MiniPET-3 scanner, providing complementary simulated and realistic low-count settings for performance assessment. The results demonstrate that incorporating attention into the LPD framework improves reconstruction fidelity and robustness compared to conventional CNN-based designs, while also highlighting the advantages of explicit cross-domain information exchange.

## 2 Materials and methods

In this section, we first review the standard LPD reconstruction algorithm, and then introduce multiple transformer-integrated LPD architectures, each designed to enhance the modeling of long-range dependencies and cross-domain interactions in PET reconstruction.

### 2.1 The LPD reconstruction algorithm

The LPD algorithm [30] is an unrolled iterative reconstruction framework that integrates forward and backward projection operations with learnable update operators in both the image (primal) and sinogram (dual) domains.

Formally, we define two families of learnable operators:

$$\Lambda_{\gamma_{i}}^{i}\;:\;\underset{i+1\;\text{times}}{\underbrace{X\times X\times \ldots \times X}}\;\to \;X,$$ (1)

$$\Xi_{\phi_{i}}^{i}\;:\;\underset{i+1\;\text{times}}{\underbrace{Y\times Y\times \ldots \times Y}}\;\to \;Y,$$ (2)

where $\gamma_{i}$ and $\phi_{i}$ denote the learnable parameters at iteration i, X denotes the image (primal) domain, and Y denotes the sinogram (dual) domain. Both X and Y are typically Hilbert spaces.

Algorithm 1 summarizes the iterative update process, where T denotes the forward projection operator, $T^{*}$ denotes its adjoint (backprojection), f∈X represents the image estimate, and g∈Y represents the corresponding sinogram. In short, at each iteration, the LPD algorithm updates the dual variables via $\Xi_{\phi_{i}}^{i}$, applies the backprojection operator $T^{*}$ to map them into the image domain, updates the primal variables via $\Lambda_{\gamma_{i}}^{i}$, and applies the forward projection operator T (except in the final iteration). Residual connections are incorporated into both $\Lambda_{\gamma_{i}}^{i}$ and $\Xi_{\phi_{i}}^{i}$ to allow information from all previous iterations in the same domain to propagate unchanged, effectively creating a memory mechanism.

Algorithm 1 **Learned Primal-Dual (LPD) Algorithm.**

**Given:** Initial sinogram $g^{0}$, Forward projection T, Number of iterations N

$g^{1}\leftarrow \Xi_{\phi_{0}}^{0}(g^{0})$

$f^{1}\leftarrow \frac{\Lambda_{\gamma_{0}}^{0}(T^{*}(g^{1}))}{\|T{\|}^{2}}$

**for** i=1,…,N **do**

 $g^{i+1}\leftarrow \Xi_{\phi_{i}}^{i}(T(f^{i}),g^{i},\ldots ,g^{0})$

 $f^{i+1}\leftarrow \frac{\Lambda_{\gamma_{i}}^{i}(T^{*}(g^{i+1}),f^{i},\ldots ,f^{0})}{\|T{\|}^{2}}$

**end** **for**

**return** $f^{i+1}$

### 2.2 The proposed transformer-based LPD reconstruction algorithms

In this section, we present our novel transformer-based LPD architectures, designed to improve PET image reconstruction by effectively incorporating global feature representations.

#### 2.2.1 Dual-domain stacked transformer-based LPD algorithm

**Figure 1:**
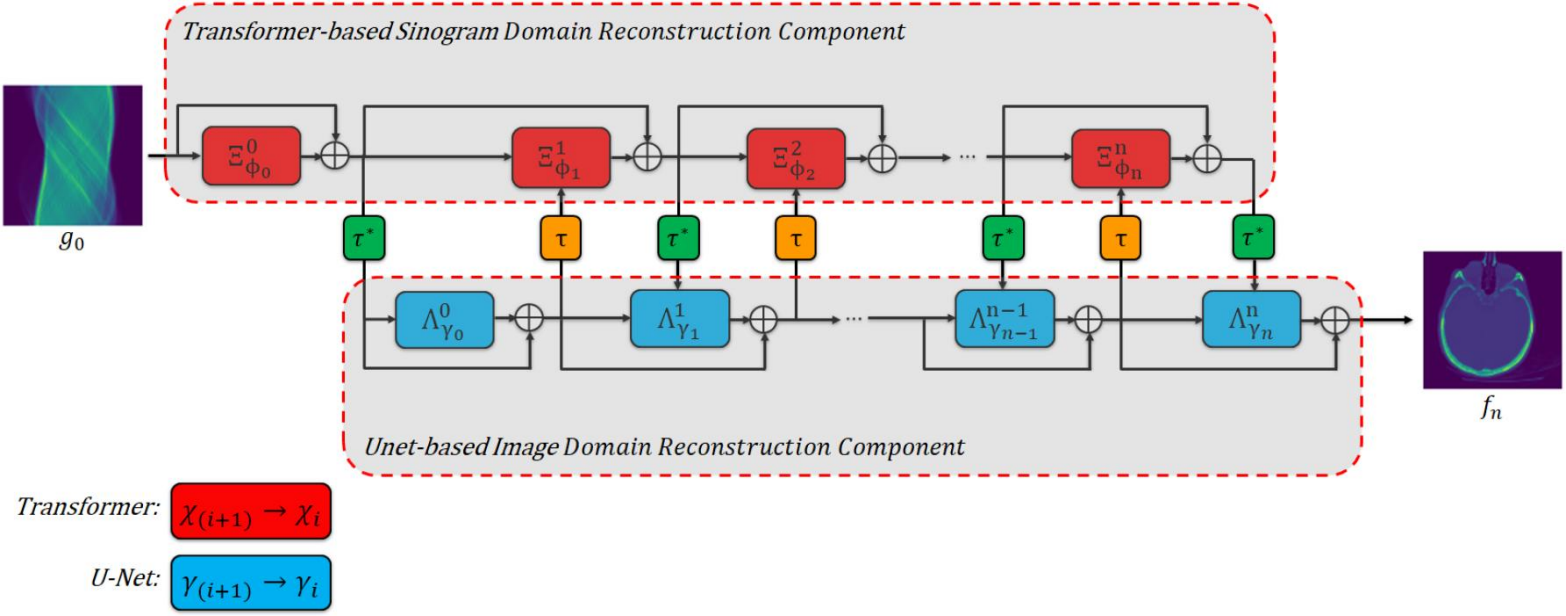
The proposed Dual-Domain Stacked Transformer-based LPD architecture. Multiple arrows converging on the same block represent channel-wise concatenation. For clarity, memory components have been excluded from this figure.

We first introduce the Dual-Domain Stacked Transformer-Based LPD algorithm. As illustrated in Figure 1, this architecture comprises two main components: a transformer-based sinogram domain reconstruction module (dual domain) and a UNet-based image domain reconstruction module (primal domain). The sinogram domain component incorporates our proposed Sinogram Sinusoidal-Structure Transformer (SSST) denoisers [22], designed to reduce noise by leveraging the knowledge of the sinusoidal patterns of the sinogram data given by the acquisition geometry. In the image domain UNet-based denoisers are deployed.

To create an effective embedding for a Transformer, the sinogram must either be down-sampled or divided into sub-problems where attention can be applied effectively. Traditional down-sampling methods, such as square patching or pooling, assume feature locality within the sinogram domain, which may not be appropriate. Instead, we exploit the fact that the acquisition geometry is known and we forward-project patches from the image domain to identify the corresponding sinogram pixels to include in a subset (token). The weighted outputs are then aggregated to reconstruct the sinogram from these curves. For embedding, the values along each curve, including their channel information, are mapped to a d-dimensional vector through a linear layer. A learnable positional encoding is then added to these embedded tokens, which are subsequently refined by a Transformer block. To reconstruct the sinogram, the updated tokens are projected back to the original dimensions of their respective sinusoidal curves using another linear layer. The final output is obtained by weighting each curve according to its activation level and adding it to the initial sinogram. Formally, the indices obtained from the forward projection of a patch $p_{i,j}$ are defined as:

$$I_{i,j}=\left\{(k,l)\;:\;{\left(Tp_{i,j}\right)}_{k,l}>0\right\}.$$ (3)

The image-space masks $p_{i,j}$ are non-overlapping rectangles of size $\left(P_{h},P_{w}\right)$ pixels, with a constant value of one, while the remaining values of the image are zero. The corresponding sinogram values extracted from g are given by:

$$z_{i,j}=\left\{g_{k,l}\;:\;(k,l)\in I_{i,j}\right\},$$ (4)

where k,l are index coordinates in the sinogram. The resulting sinusoidal curves $z_{i,j}$ are then embedded using a linear layer and subsequently updated by a Transformer as follows: ?>

$$z_{i,j}^{emb}=\text{Linear}(z_{i,j})+P$$ (5)

$$Q,\;K,\;V=\text{Linear}(z_{i,j}^{emb}),\;\text{Linear}(z_{i,j}^{emb}),\;\text{Linear}(z_{i,j}^{emb})$$ (6)

$$z_{i,j}^{emb}=\text{LayerNorm}(z_{i,j}^{emb}+\text{MultiHead}(Q,K,V))$$ (7)

$$z_{i,j}^{emb}=\text{LayerNorm}(z_{i,j}^{emb}+\text{MLP}(z_{i,j}^{emb})),$$ (8)

where P is a learnable positional encoding. The final reconstruction update is given by:

$$z_{i,j}=\text{Linear}(z_{i,j}^{emb})$$ (9)

$$g_{k,l}=g_{k,l}+\sum_{i,j}(z_{i,j}{)}_{k,l}(W_{i,j}{)}_{k,l},$$ (10)

with activation weights $(W_{i,j}{)}_{k,l}$ defined as:

$$(W_{i,j}{)}_{k,l}={\left(Tp_{i,j}\right)}_{k,l}.$$ (11)

By integrating the proposed Sinogram Sinusoidal-Structure Transformer (SSST) into the LPD architecture, we introduce the Dual-Domain Stacked Transformer-based LPD algorithm, summarized in Algorithm 2.

Algorithm 2: **Dual-Domain Stacked Transformer-based LPD Algorithm**

**Given:** Initial sinogram $g^{0}$, Patch size $\left(P_{h},P_{w}\right)$, Forward Projection T, Number of iterations N

$f^{0}\leftarrow T^{*}(g^{0})$

Initialize an empty list of Patch-isolated images: P←∅

Let *H* and *W* be the height and width of $f^{0}$, respectively

**for** i=0 **to** $\lfloor +;\frac{H}{P_{h}}\rfloor -1$ **do**

 **for** j=0 **to** $\lfloor +;\frac{W}{P_{w}}\rfloor -1$ **do**

  Initialize matrix $p_{i,j}$ of dimensions H×W with zeros

  Set $p_{i,j}[i\cdot P_{h}:(i+1)\cdot P_{h},j\cdot P_{w}:(j+1)\cdot P_{w}]\leftarrow f^{0}[i\cdot P_{h}:(i+1)\cdot P_{h},j\cdot P_{w}:(j+1)\cdot P_{w}]$

  Add $p_{i,j}$ to the list of Patch-isolated images: $P\leftarrow P\cup \{p_{i,j}\}$

 **end** **for**

**end** **for**

**for** each Patch-isolated images $p_{i,j}$ in P **do**

 Initialize set of indices $I_{i,j}\leftarrow \emptyset$

 **for** each index (k,l) in $T(p_{i,j})$ **do**

  **if** $(Tp_{i,j}{)}_{k,l}>0$ **then**

   $I_{i,j}\leftarrow I_{i,j}\cup \{(k,l)\}$

  **end** **if**

 **end** **for**

 Extract values $z_{i,j}\leftarrow \{g_{k,l}^{0}:(k,l)\in I_{i,j}\}$

 Embed $z_{i,j}^{emb}\leftarrow Linear(z_{i,j})+P$

 Compute $Q\leftarrow Linear(z_{i,j}^{emb})$, $K\leftarrow Linear(z_{i,j}^{emb})$, $V\leftarrow Linear(z_{i,j}^{emb})$

 Update $z_{i,j}^{emb}\leftarrow LayerNorm(z_{i,j}^{emb}+MultiHead(Q,K,V))$

 Apply MLP: $z_{i,j}^{emb}\leftarrow LayerNorm(z_{i,j}^{emb}+MLP(z_{i,j}^{emb}))$

 Final output: $z_{i,j}\leftarrow Linear(z_{i,j}^{emb})$

 Compute weights: $(W_{i,j}{)}_{k,l}\leftarrow (Tp_{i,j}{)}_{k,l}$

**end** **for**

**for** each (k,l) in $I_{i,j}$ **do**

 Update $g_{k,l}^{0}\leftarrow g_{k,l}^{0}+\sum_{i,j}(z_{i,j}{)}_{k,l}\times (W_{i,j}{)}_{k,l}$

**end** **for**

Set $g^{1}\leftarrow g^{0}$ (i.e.,$\Xi_{\phi_{0}}^{0}(g^{0})$)

$f^{1}\leftarrow \Lambda_{\gamma_{0}}^{0}(T^{*}(g^{1}))/||T|{|}^{2}$

**for** i=1,…,N **do**

 $g^{i+1}\leftarrow \Xi_{\phi_{i}}^{i}(T(f^{i}),g^{i},\ldots ,g^{0})$

 $f^{i+1}\leftarrow \Lambda_{\gamma_{i}}^{i}(T^{*}(g^{i+1}),f^{i},\ldots ,f^{0}))/||T|{|}^{2}$

**end** **for**

**return** $f^{i+1}$

#### 2.2.2 Dual-domain Restormer-based LPD algorithm

The Restormer architecture [33] integrates convolutional operations for local feature extraction with attention mechanisms for modeling global context. Motivated by this, we explore the Restormer as an alternative to both the UNet and Dual-Domain Stacked Transformer architectures.

Within each Transformer block of the Restormer, embeddings are generated using a 1 × 1 convolutional layer that triples the number of channels, deviating from the conventional linear embedding approach. The Restormer replaces standard linear layers for computing the Key, Query, and Value matrices with depth-wise convolutions using a 3 × 3 kernel. These depth-wise convolutions are particularly effective at capturing channel-wise features rather than spatial ones. Multi-head attention is then applied along the channel dimension, differing from the typical approach of flattening spatial dimensions for attention calculation. Furthermore, instead of a standard MLP, each Transformer block incorporates a Gated Depth-wise Convolution (Dconv) Feed-Forward Network. Convolutional layers handle the up-sampling and down-sampling operations, doubling or halving the number of channels as needed. For further details, see [33]. Similar to the previous variants, the Restormer-based LPD operates in a dual-domain iterative framework, where each iteration consists of updates in both the projection (sinogram) domain and the image domain.

#### 2.2.3 Dual-domain UNet-based LPD algorithm incorporating a cross-attention mechanism

**Figure 2:**
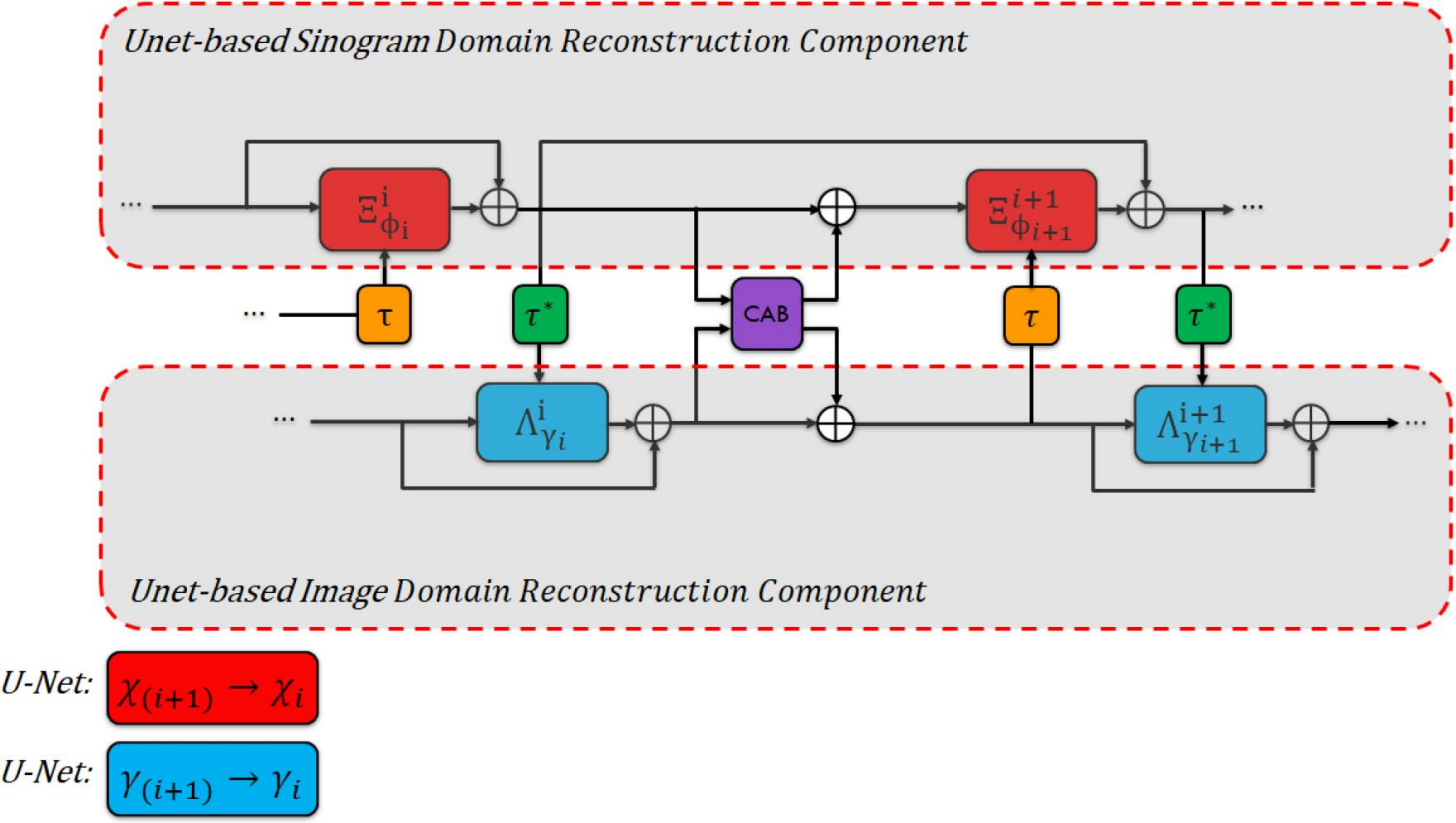
The proposed Dual-Domain UNet-based LPD architecture incorporating a Cross-Attention mechanism. Multiple arrows going into the same module except for CAB, indicate channel-wise concatenation. For clarity, memory components have been excluded from this figure.

In an ideal scenario, an image and the corresponding sinogram contain the same information. Attention could then be used to characterise how well a candidate image fits the measured projection data. We therefore propose here a LPD architecture (see Figure 2) that incorporates Cross-Attention blocks between the primal (image) and dual (sinogram) channels of the LPD algorithm.

As outlined in Algorithm 3, each Cross Attention Block (CAB) takes both sinogram and image inputs. In the image domain, patches are extracted using the standard patching process, while in the sinogram domain, each patch corresponds to the forward projection of its associated image patch and is embedded into the same dimensional space. The Queries, Keys, and Values for each domain are computed separately through linear layers. Denoting the primal Queries, Keys, and Values as $Q_{p}$, $K_{p}$, $V_{p}$, and the dual Queries, Keys, and Values as $Q_{d}$, $K_{d}$, $V_{d}$, the cross-attention computations are defined as: ?>

$${\text{att}}_{p}=\text{MultiHead}(Q_{d},K_{p},V_{p})),$$ (12)

$${\text{att}}_{d}=\text{MultiHead}(Q_{p},K_{d},V_{d})).$$ (13)

The resulting Attention outputs for each domain are added to their respective embedded tokens, followed by layer normalization and processing through an MLP. The MLP outputs are normalized again and added back to the input sinogram and image representations.

Finally, during the scatter-back reconstruction step in the sinogram domain, the term indices[:, :, 2] denotes the projection-derived weighting mask obtained from the forward projection of each image patch, used to modulate the contribution of each token during the scatter-back reconstruction. This weighting is applied when mapping the updated token representations back to their corresponding spatial locations in the sinogram domain.

While restricting information exchange between domains within LPD may act as a form of regularization, allowing this exchange without the constraints of projection operations can provide additional benefits, enhancing the reconstruction process.

### 2.3 Maximum likelihood expectation maximization (MLEM)

As a conventional baseline, we employed the Maximum Likelihood Expectation Maximization (MLEM) algorithm [34] for image reconstruction. MLEM iteratively updates the image estimate by maximizing the Poisson likelihood of the measured projection data. Assuming a Poisson model, the measured data y are given by:

y∼Poisson(Ax+r), (14)

where x is the unknown image, A is the system matrix, and r represents additive background events (e.g., scatter and randoms). The MLEM update rule is given by:

$$x^{\left(k+1\right)}=x^{\left(k\right)}\cdot \frac{A^{T}\left(\frac{y}{Ax^{\left(k\right)}+r}\right)}{A^{T}1}$$ (15)

where $A^{T}$ denotes the backprojection operator and 1 is a vector of ones.

In this study, the MLEM reconstruction was performed for 20 iterations starting from a uniform initialization. The system matrix models the acquisition geometry of the scanner, and a Gaussian point spread function (PSF) (5×5, σ=2) was used to model the system resolution.

### 2.4 Reconstruction evaluation framework

#### 2.4.1 Quantitative metrics (MSE, PSNR, SSIM)

The reconstruction quality is evaluated using Mean Squared Error (MSE), Peak Signal-to-Noise Ratio (PSNR), and Structural Similarity Index Measure (SSIM). The MSE is defined as:

$$\mathrm{MSE}=\frac{1}{N}\sum_{i=1}^{N}(x_{i}-x_{i}^{\mathrm{ref}}{)}^{2},$$ (16)

where $x_{i}$ and $x_{i}^{\mathrm{ref}}$ denote the reconstructed and reference pixel values, respectively. PSNR is computed as:

$$\mathrm{PSNR}=10\log_{10}\left(\frac{I_{\max}^{2}}{\mathrm{MSE}}\right),$$ (17)

where $I_{\max}$ is the maximum possible intensity value. SSIM measures perceptual similarity between the reconstructed and reference images and is defined as:

$$\mathrm{SSIM}(x,x^{\mathrm{ref}})=\frac{\left(2\mu_{x}\mu_{x^{\mathrm{ref}}}+C_{1})(2\sigma_{xx^{\mathrm{ref}}}+C_{2}\right)}{\left(\mu_{x}^{2}+\mu_{x^{\mathrm{ref}}}^{2}+C_{1})(\sigma_{x}^{2}+\sigma_{x^{\mathrm{ref}}}^{2}+C_{2}\right)},$$ (18)

where $\mu_{x}$ and $\mu_{x^{\mathrm{ref}}}$ denote the mean intensities, $\sigma_{x}^{2}$ and $\sigma_{x^{\mathrm{ref}}}^{2}$ are the variances, $\sigma_{xx^{\mathrm{ref}}}$ is the covariance between the images, and $C_{1}$, $C_{2}$ are small constants to stabilize the division.

Algorithm 3: **Cross Attention Block (CAB).**

**Given:** Primal input *x*, Dual input *y*, patch size *p*, Indices

$x_{emb}\leftarrow$ Linear embedding of *x* with patch size p×p

$y_{emb}\leftarrow$ Linear embedding of extracted patches from *y* using indices

$x_{emb}\leftarrow x_{emb}+$ Positional Encodings for Primal Input

$y_{emb}\leftarrow y_{emb}+$ Positional Encodings for Dual Input

$Q_{p},K_{p},V_{p}\leftarrow$ Linear projections of $x_{emb}$ {Primal attention queries, keys, values}

$Q_{d},K_{d},V_{d}\leftarrow$ Linear projections of $y_{emb}$ {Dual attention queries, keys, values}

**Attention Computation:**

$attention_{p}\leftarrow$ softmax $\left(\frac{Q_{d}\cdot K_{p}^{T}}{\sqrt{head\_dim}}\right)\cdot V_{p}$

$attention_{d}\leftarrow$ softmax $\left(\frac{Q_{p}\cdot K_{d}^{T}}{\sqrt{head\_dim}}\right)\cdot V_{d}$

$x_{emb}\leftarrow$ LayerNorm $\left(x_{emb}+attention_{p}\right)$

$y_{emb}\leftarrow$ LayerNorm $\left(y_{emb}+attention_{d}\right)$

$x_{emb}\leftarrow$ LayerNorm $\left(x_{emb}+MLP(x_{emb})\right)$

$y_{emb}\leftarrow$ LayerNorm $\left(y_{emb}+MLP(y_{emb})\right)$

$x_{\;full}\leftarrow$ Unembed and reshape $x_{emb}$ back to original primal size

$y_{c}\leftarrow$ Unembed and reshape $y_{emb}$ using indices

$y_{out}\leftarrow y[:,-1,:,:].unsqueeze(1)$

$y_{\;full}\leftarrow zeros\_like(y_{out})$

$y_{\;full}[:,:,indices[:,:,0],indices[:,:,1]]\leftarrow y_{c}\cdot indices[:,:,2]$

$x\leftarrow x[:,-1,:,:].unsqueeze(1)+x_{\;full}$

$y\leftarrow y_{out}+y_{\;full}$

**return** x,y

#### 2.4.2 Qualitative criteria (unrolling visualization and variance analysis)

1. **Unrolling visualization:** To analyze the behavior of the learned iterative reconstruction, we visualize the intermediate outputs of the unrolled network across iterations. Specifically, each stage of the unrolled LPD network corresponds to one iteration, producing a progressively refined image estimate. By extracting and displaying these intermediate reconstructions, we assess how different architectures propagate and refine image features throughout the iterative process.
2. **Variance analysis:** To assess model robustness, we compute and visualize the pixel-wise variance of reconstructed images obtained from multiple noise realizations of the Shepp–Logan phantom at a noise level of 0.3. This analysis highlights spatial regions that are most sensitive to noise and allows qualitative assessment of reconstruction stability across different methods.

#### 2.4.3 Capacity-controlled baseline experiment

To investigate whether the performance improvements of the proposed Transformer-based LPD models are primarily driven by increased model capacity, we introduce an additional capacity-controlled UNet-based LPD baseline. In this experiment, referred to as UNet-based LPD (4 Iterations), the number of unrolled iterations in the UNet-LPD framework is increased from 3 to 4, resulting in increased representational capacity. Further increasing the number of iterations to more closely match the parameter scale of the Transformer-based models was not feasible due to GPU memory limitations. All other architectural components, training settings, and evaluation protocols are kept identical to those of the original UNet-LPD model, ensuring a controlled comparison. This setup enables the assessment of performance differences under increased capacity while preserving the same iterative reconstruction framework.

## 3 Results

### 3.1 Datasets

To train the proposed architectures, we used synthetically generated 3D images of size 147×147×d, where d (image depth) was chosen according to available memory. Each image contains a random number of ellipsoids, with the count sampled from a Poisson distribution with rate parameter λ=10. The center of each ellipsoid was drawn uniformly within the image dimensions, the axis lengths followed an exponential distribution with rate parameter λ=0.3, and the orientation angles were sampled uniformly from the range [0,2π). The activity concentration of each ellipsoid was sampled uniformly from [0,1].

**Figure 3:**
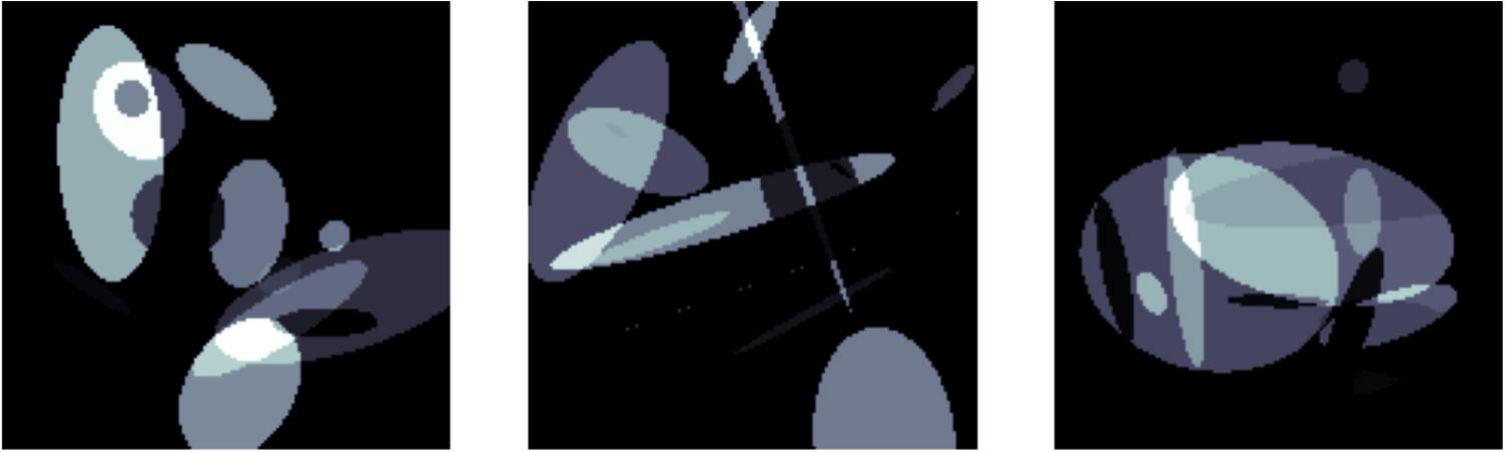
Samples of synthetic images used for training.

To create hollow structures, the same procedure was repeated, but with negative activity concentrations. In this case, the axis lengths were drawn from an exponential distribution with rate parameter λ=0.15. After generating the structures, the absolute value of each pixel was taken to ensure all voxel intensities are non-negative. The resulting images served as the ground truth data for our experiments. Examples of these ground truth volumes are shown in Figure 3.

To approximate the characteristics of the target preclinical system (MiniPET-3) [35, 36], our in-house PET scanner dedicated to small-animal imaging, a Gaussian blur with a kernel size of 5 and σ=2 was applied to the synthetic images. This setting reflects the use of fludeoxyglucose in the preclinical PET system [36], which exhibits an average positron range of 0.6 mm and a maximum range of 2.4 mm; in this particular case, 1 mm corresponds to approximately 1.8 pixels.

**Figure 4:**
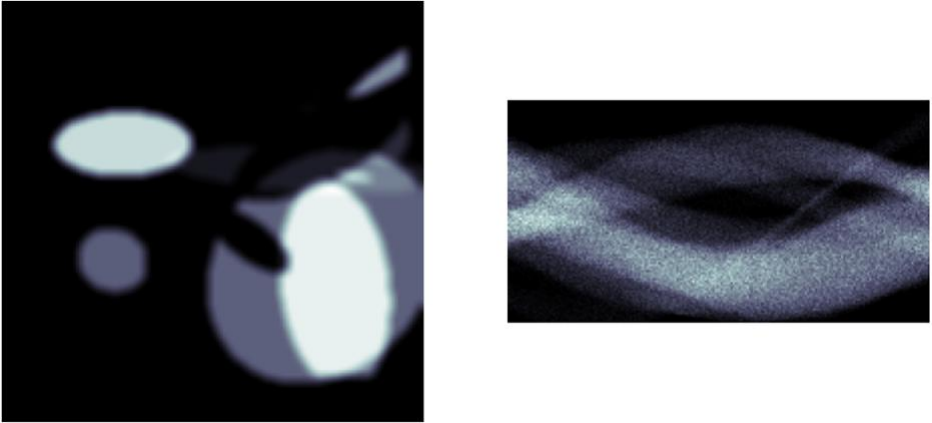
Example of a blurred synthetic image alongside its corresponding noisy sinogram.

**Figure 5:**
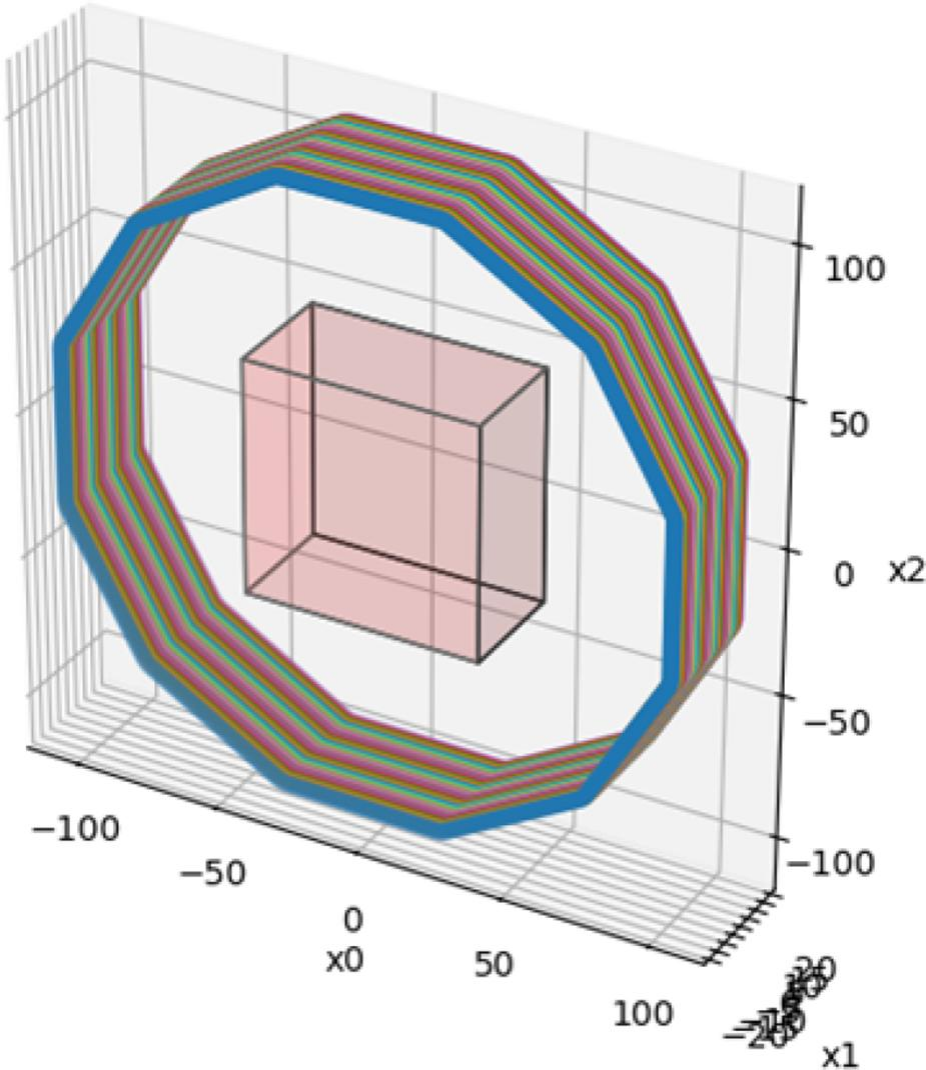
The MiniPET-3 scanner geometry and acquisition volume utilized in our PET image reconstruction experiments.

**Figure 6:**
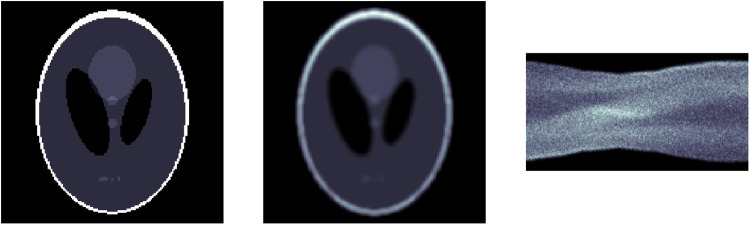
Slice of the Shepp–Logan phantom, shown with its blurred training example and corresponding noisy sinogram.

The blurred training images were forward projected using parallelproj [37] to generate sinograms. Poisson noise was then added by sampling with a rate parameter equal to the pixel value divided by the noise level, followed by multiplication by the same noise level. The noise level was used as a relative scaling factor controlling the Poisson rate, serving as a surrogate for varying count statistics rather than a direct calibration to physical scanner quantities such as total counts or Noise Equivalent Counts (NEC). For each training example, the noise level was sampled uniformly from the interval [0.1,1.2] to simulate a range of low- to high-noise acquisition conditions within the training distribution. An example of the resulting training data is shown in Figure 4. The MiniPET-3 system, modeled as a cylindrical scanner with 12 detector modules each containing 35 × 35 crystals (Figure 5), was replicated in parallelproj by configuring the acquisition geometry to closely match the target system, thereby ensuring that the synthetic data generation process remains well-aligned with the preclinical setup. For evaluation, a synthetic test set was created using 35 slices of the Shepp–Logan phantom. The same transformations used in training were applied, with varying noise levels. A representative example from the test set is shown in Figure 6.

**Figure 7:**
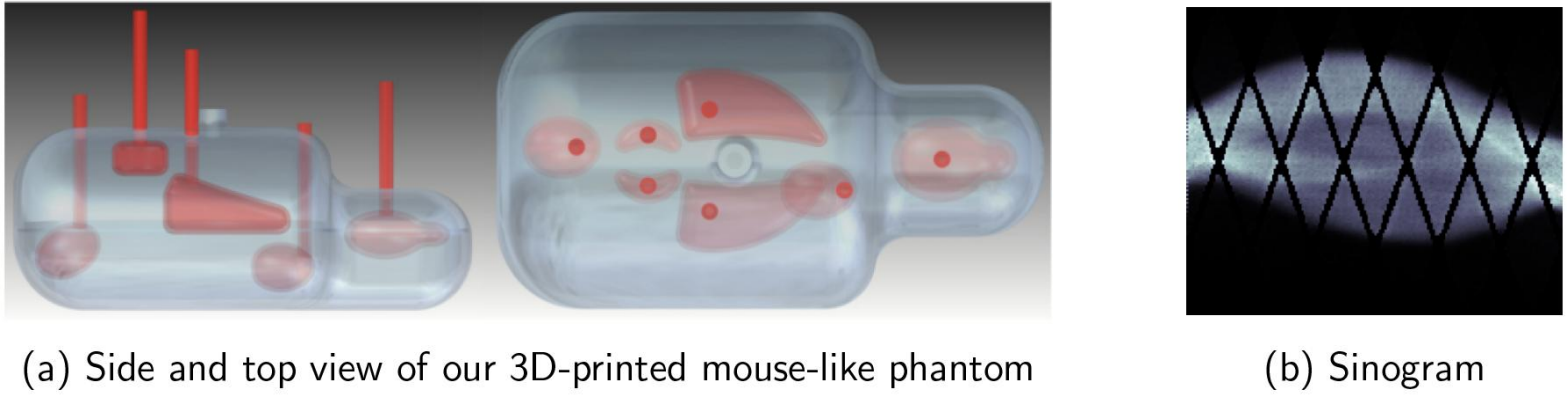
Our 3D-printed mouse-like phantom **(a)** and an example sinogram from our MiniPET-3 scanner **(b)**.

For the experimental test set, we used a mouse-like phantom that had been previously 3D-printed and scanned in our earlier work [12], as illustrated in Figure 7a. The activity concentrations assigned to the organs, for two different configurations, are listed in Table 1. This dataset comprises sinograms derived from images of size 147×147×35, with activity measurements acquired every minute over a one-hour period. An example sinogram from the target system is shown in Figure 7b.

**Table 1:** Activity concentrations for the test phantom for two different measurements.

Body$\left[\frac{\text{MBq}}{\text{mL}}\right]$	Brain$\left[\frac{\text{MBq}}{\text{mL}}\right]$	Heart$\left[\frac{\text{MBq}}{\text{mL}}\right]$	Lungs$\left[\frac{\text{MBq}}{\text{mL}}\right]$	Kidneys$\left[\frac{\text{MBq}}{\text{mL}}\right]$	Bladder$\left[\frac{\text{MBq}}{\text{mL}}\right]$
0.5	1.1	0.1	0.15	0.8	1.3
0.4	1.1	0.1	0.07	0.9	out of FOV

### 3.2 Implementation and parameter setting

We trained all models using MSE loss, Gaussian initialization with σ=0.02, and the AdamW optimizer with a weight decay of 0.01. The learning rate was adjusted using the ReduceLROnPlateau strategy, and training was guided by early stopping with best-metric tracking, allowing each model to stop automatically at the point of best generalization. The initial learning rate was set to $5\times 10^{-4}$ for all models except the Restormer-based LPD, which started at $2.5\times 10^{-4}$. We report MSE, Peak Signal-to-Noise Ratio (PSNR) and Structural Similarity Index Measure (SSIM) as metrics for evaluating reconstruction quality. The Wilcoxon signed-rank test [38] was also performed to assess the statistical significance of differences between reconstructed images produced by the proposed methods and the baseline algorithm. A p-value below 0.0001 was considered indicative of a statistically significant difference. Additionally, visual inspection was used as a qualitative assessment.

To establish a baseline, we employed the UNet-based LPD model from previous work [12], trained with three iterations and ReLU activation functions. The proposed Dual-Domain Stacked Transformer-based LPD model employs three Transformers within each dual block, each using 8 attention heads with an embedding dimension of 64 per head, over three iterations. The primal channel incorporates U-Nets. The Dual-Domain Restormer-based LPD uses three iterations, with both primal and dual channels employing Restormers. Each block (excluding the refinement block) contains 2, 3, 3, 4, 3, 3, and 2 Transformers, with head counts of 1, 2, 4, 4, 4, 2, and 1, respectively. The refinement block consists of four single-head Transformers. Finally, the proposed UNet-based LPD + CAB model was implemented with three iterations. Each CAB consists of two Transformers (one per domain) with query swapping, each having 8 heads and an embedding dimension of 64. Two CABs were inserted between iterations 1–2 and 2–3, using a patch size of 7×7. All models were trained on images with dimensions 147×147×35. The models and their respective parameter counts are listed in Table 2, together with training and inference times, to evaluate both computational complexity and runtime performance.

**Table 2:** Model characteristics of baseline and proposed LPD architectures.

Algorithm	Number of parameters	Training time (h)	Inference time (s)
UNet-based LPD (3 Iterations)	**12,862,278**	**52**	**1.5610**
UNet-based LPD (4 Iterations)	17,150,952	68	2.2653
Dual-Domain Stacked Transformer-based LPD	29,429,100	65	1.8150
Dual-Domain Restormer-based LPD	23,248,584	73	18.6941
UNet-based LPD+CAB	23,131,886	56	1.6587

Bold values indicate the best results among the compared methods.

The training dataset for all LPD models consisted of 200 synthetic volumes, each comprising 35 slices, generated on-the-fly using a random ellipsoid-based phantom generator. The networks were trained for 200 epochs with a batch size of 1, which was selected due to GPU memory constraints associated with the 3D data and the unrolled architecture. All LPD models employed 3 unrolled iterations. The preprocessing of experimental data (sinograms) consists solely of spatial resizing to match the network input dimensions and clipping of negative values. The proposed networks were implemented in Python using the PyTorch framework and trained on a server equipped with an NVIDIA Tesla K80 GPU (11 GB memory). The ODL library [39] was employed to generate ellipsoids, while parallelproj [37] was used to define the projector geometry and perform forward and backward projections, leveraging GPU acceleration for computational efficiency.

### 3.3 Experimental results

#### 3.3.1 Synthetic data

The reconstruction performance on the Shepp-Logan phantom across four noise levels (n=0.3, 0.7, 1.0 and 1.5) is summarized in Tables 3–5. While noise levels of 0.3, 0.7, and 1.0 lie within the training distribution, we additionally evaluated robustness beyond the training regime by including an out-of-distribution setting with a higher noise level of 1.5, corresponding to a lower-count regime than that observed during training. Compared to the UNet-based LPD baseline, the Dual-Domain Stacked Transformer-based LPD improves MSE and PSNR but exhibits lower SSIM, suggesting more accurate intensity reconstruction at the expense of some structural detail. The Restormer-based model consistently yields improvements in MSE and PSNR across all noise levels and achieves higher SSIM at low and moderate noise levels (0.3 and 0.7), while showing slightly lower SSIM at higher noise levels (1.0 and 1.5). The UNet-based LPD with Cross-Attention also improves MSE and PSNR over the baseline, with a slight reduction in SSIM, indicating a trade-off between pixel-wise accuracy and structural similarity. The classical MLEM reconstruction yields the lowest performance across all metrics, highlighting the limitations of traditional reconstruction methods under these noise conditions and the advantages of learned LPD approaches.

**Table 3:** MSE values for the synthetic test set at four different noise levels.

Algorithm	MSE (Mean±Std)			
	*n* = 0.3	*n* = 0.7	*n* = 1.0	*n* = 1.5
UNet-based LPD (Baseline)	$5.42\times 10^{-3}$ ± $15\times \times 10^{-5}$	$5.98\times 10^{-3}$ ± $23\times 10^{-5}$	$6.45\times 10^{-3}$ ± $78\times 10^{-5}$	$7.88\times 10^{-3}$ ± $10\times 10^{-4}$
Stacked transformer-based LPD	$4.72\times 10^{-3}$ ± $11\times 10^{-5a}$	$5.05\times 10^{-3}$ ± $19\times 10^{-5a}$	$5.19\times 10^{-3}$ ± $62\times 10^{-5a}$	$5.73\times 10^{-3}$ ± $96\times 10^{-5a}$
Restormer-based LPD	**3.43 × 10^−3^ ± 10 × 10^−5a^**	$4.38\times 10^{-3}$ ± $15\times 10^{-5a}$	$5.04\times 10^{-3}$ ± $57\times 10^{-5a}$	$5.44\times 10^{-3}$ ± $91\times 10^{-5a}$
UNet-based LPD+CAB	$3.89\times 10^{-3}$ ± $20\times 10^{-5a}$	**3.99 × 10^−3^ ± 33 × 10^−5a^**	**4.21 × 10^−3^ ± 96 × 10^−5a^**	**4.75 × 10^−3^ ± 39 × 10^−5a^**
MLEM	$1.46\times 10^{-2}$ ± $17\times 10^{-4}$	$1.57\times 10^{-2}$ ± $31\times 10^{-4}$	$1.64\times 10^{-2}$ ± $67\times 10^{-4}$	$2.22\times 10^{-2}$ ± $98\times 10^{-4}$

${}^{a}$Indicates statistically significant improvement compared to the UNet-based LPD baseline based on the Wilcoxon signed-rank test (P<0.0001).

Bold values indicate the best results among the compared methods.

**Table 4:** PSNR values for the synthetic test set at four different noise levels.

Algorithm	PSNR(dB) (Mean±Std)			
	*n* = 0.3	*n* = 0.7	*n* = 1.0	*n* = 1.5
UNet-based LPD (Baseline)	21.103 ± 0.273	20.989 ± 0.290	20.763 ± 0.317	19.750 ± 0.368
Stacked transformer-based LPD	22.729 ± 0.115${}^{a}$	22.275 ± 0.213${}^{a}$	21.944 ± 0.202${}^{a}$	20.125 ± 0.369${}^{a}$
Restormer-based LPD	**24.660 ± 0.170** ${}^{a}$	23.003 ± 0.245${}^{a}$	22.866 ± 0.282${}^{a}$	21.210 ± 0.322${}^{a}$
UNet-based LPD+CAB	23.589 ± 0.256${}^{a}$	**23.276 ± 0.270** ${}^{a}$	**22.993 ± 0.293** ${}^{a}$	**21.415 ± 0.319** ${}^{a}$
MLEM	18.444 ± 0.139	18.000 ± 0.155	17.886 ± 0.234	16.425 ± 0.276

${}^{a}$Indicates statistically significant improvement compared to the UNet-based LPD baseline based on the Wilcoxon signed-rank test (P<0.0001).

Bold values indicate the best results among the compared methods.

**Table 5:** SSIM values for the synthetic test set at four different noise levels.

Algorithm	SSIM (Mean±Std)			
	*n* = 0.3	*n* = 0.7	*n* = 1.0	*n* = 1.5
UNet-based LPD (Baseline)	0.927 ± 0.005	0.916 ± 0.008	**0.909 ± 0.015**	**0.890 ± 0.022**
Stacked transformer-based LPD	0.905 ± 0.004	0.906 ± 0.009	0.900 ± 0.025	0.807 ± 0.037
Restormer-based LPD	**0.938 ± 0.001** ${}^{a}$	**0.919 ± 0.005** ${}^{a}$	0.907 ± 0.014	0.873 ± 0.039
UNet-based LPD+CAB	0.866 ± 0.007	0.861 ± 0.012	0.851 ± 0.047	0.791 ± 0.051
MLEM	0.663 ± 0.001	0.602 ± 0.004	0.579 ± 0.010	0.520 ± 0.029

${}^{a}$Indicates statistically significant improvement compared to the UNet-based LPD baseline based on the Wilcoxon signed-rank test (P<0.0001).

Bold values indicate the best results among the compared methods.

**Figure 8:**
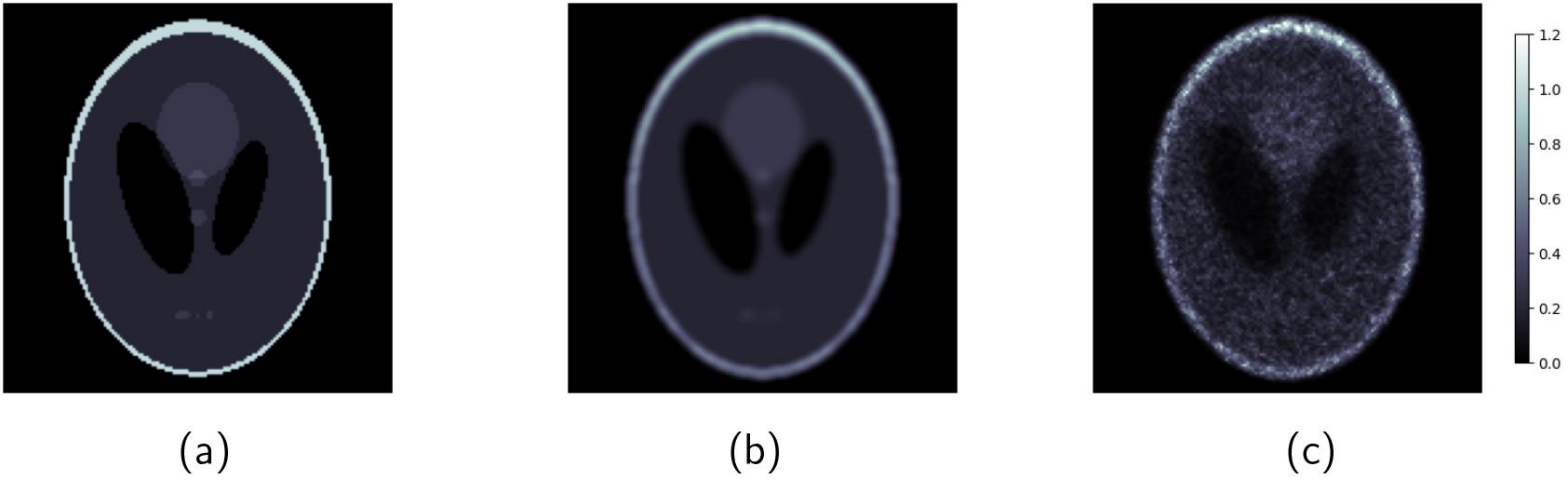
Ground truth Shepp-Logan phantom, shown both with and without Gaussian blur, alongside an MLEM reconstruction at a noise level of 0.7. **(a)** Ground truth. **(b)** Blurred ground truth. **(c)** MLEM reconstruction.

For a visual comparison on the synthetic test set, Figure 8 shows the ground truth Shepp-Logan phantom, both with and without Gaussian blur, alongside an MLEM reconstruction.

**Figure 9:**
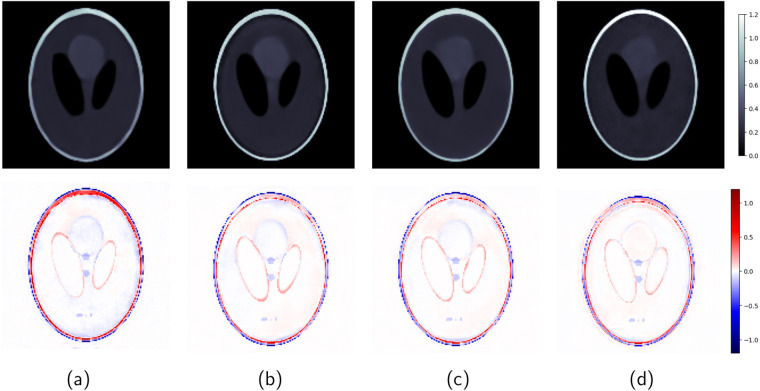
First row: reconstructed images from the test set using the baseline UNet-based LPD and all proposed transformer-based LPD models at a noise level of 0.3. Second row: corresponding difference images relative to the ground truth. Blue and red colors indicate underestimation and overestimation of pixel values with respect to the ground truth, respectively. **(a)** UNet-based LPD. **(b)** Stacked Transformer-based LPD. **(c)** Restormer-based LPD. **(d)** UNet-based LPD+CAB.

**Figure 10:**
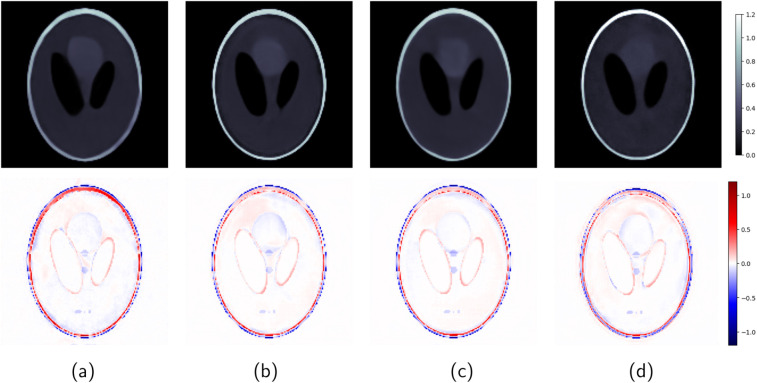
First row: reconstructed images from the test set using the baseline UNet-based LPD and all proposed transformer-based LPD models at a noise level of 0.7. Second row: corresponding difference images relative to the ground truth. Blue and red colors indicate underestimation and overestimation of pixel values with respect to the ground truth, respectively. **(a)** UNet-based LPD. **(b)** Stacked Transformer-based LPD. **(c)** Restormer-based LPD. **(d)** UNet-based LPD+CAB.

**Figure 11:**
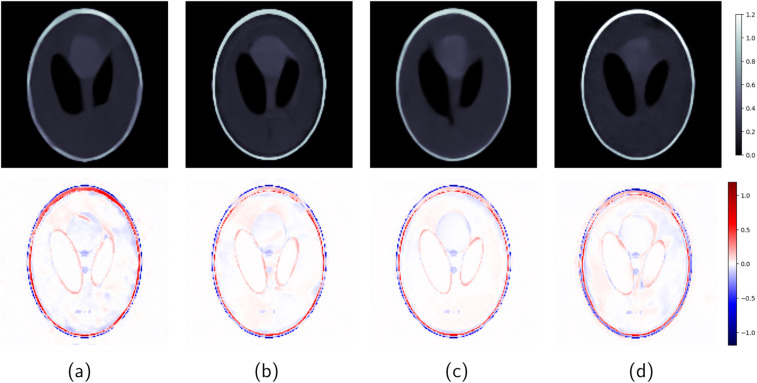
First row: reconstructed images from the test set using the baseline UNet-based LPD and all proposed transformer-based LPD models at a noise level of 1. Second row: corresponding difference images relative to the ground truth. Blue and red colors indicate underestimation and overestimation of pixel values with respect to the ground truth, respectively. **(a)** UNet-based LPD. **(b)** Stacked Transformer-based LPD. **(c)** Restormer-based LPD. **(d)** UNet-based LPD+CAB.

Figures 8–11 present a visual comparison of the classical MLEM reconstruction and all the proposed transformer-based LPD algorithms against the UNet-based LPD algorithm. In Figure 9, when comparing the reconstructions with the ground truth, the differences are subtle. For example, although the Dual-Domain Stacked Transformer-based LPD achieves higher PSNR and lower MSE than the UNet-based LPD baseline, the visual differences between their reconstructions are not immediately apparent. The Dual-Domain Restormer-based LPD provides the sharpest reconstructions, particularly in recovering the phantom’s outer shell, closely matching the non-blurred ground truth. However, none of the models, including MLEM (see Figure 8), manage to recover the very small white regions between the cavities, likely due to their challenging size and placement, compounded by Gaussian blurring effects. MLEM reconstructions appear overall blurrier and less structurally faithful, which is consistent with their weaker performance on the reported figure of merits.

In Figures 10, 11, where synthetic test data is reconstructed from noisier sinograms, degradation becomes more pronounced across all methods. The cavities become distorted, yet the shell and the contrast of the larger white regions between cavities remain relatively well preserved. Among the compared methods, UNet-based LPD+CAB maintains the highest visual quality followed by the Dual-Domain Restormer-based LPD. Both outperform the Stacked Transformer-based LPD, the UNet-based LPD, and especially the MLEM reconstructions, which exhibits the most severe blurring and structural loss.

#### 3.3.2 Preclinical data

**Figure 12:**
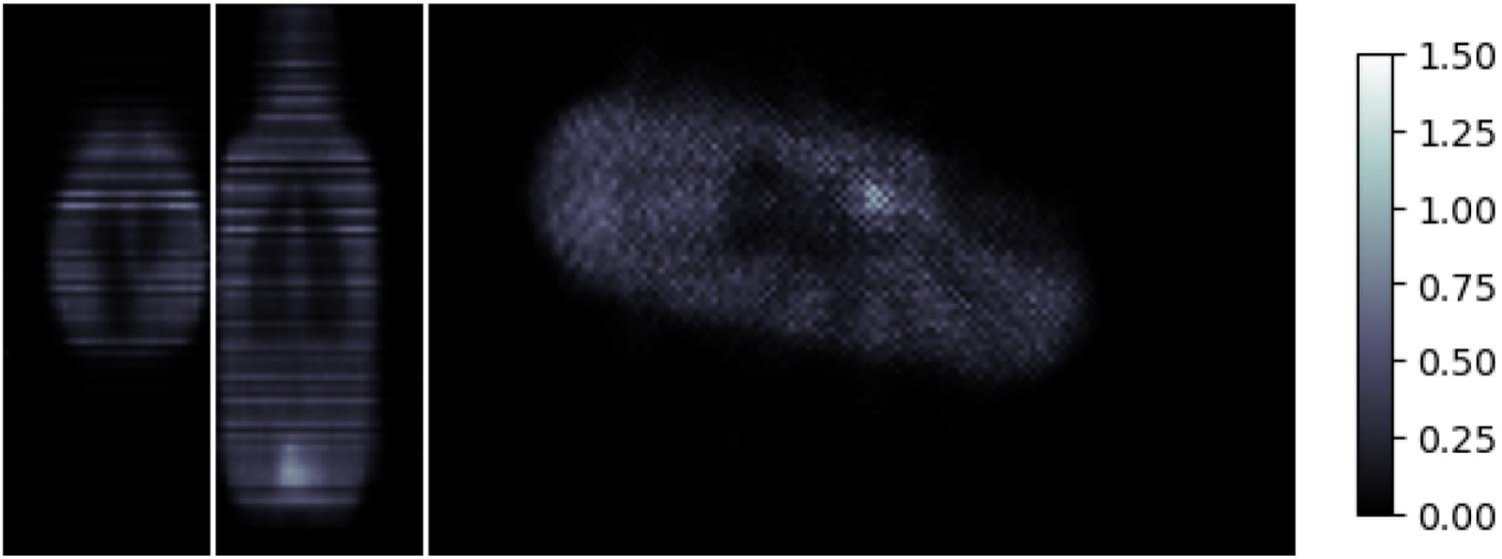
MLEM reconstruction of the mouse-like phantom.

**Figure 13:**
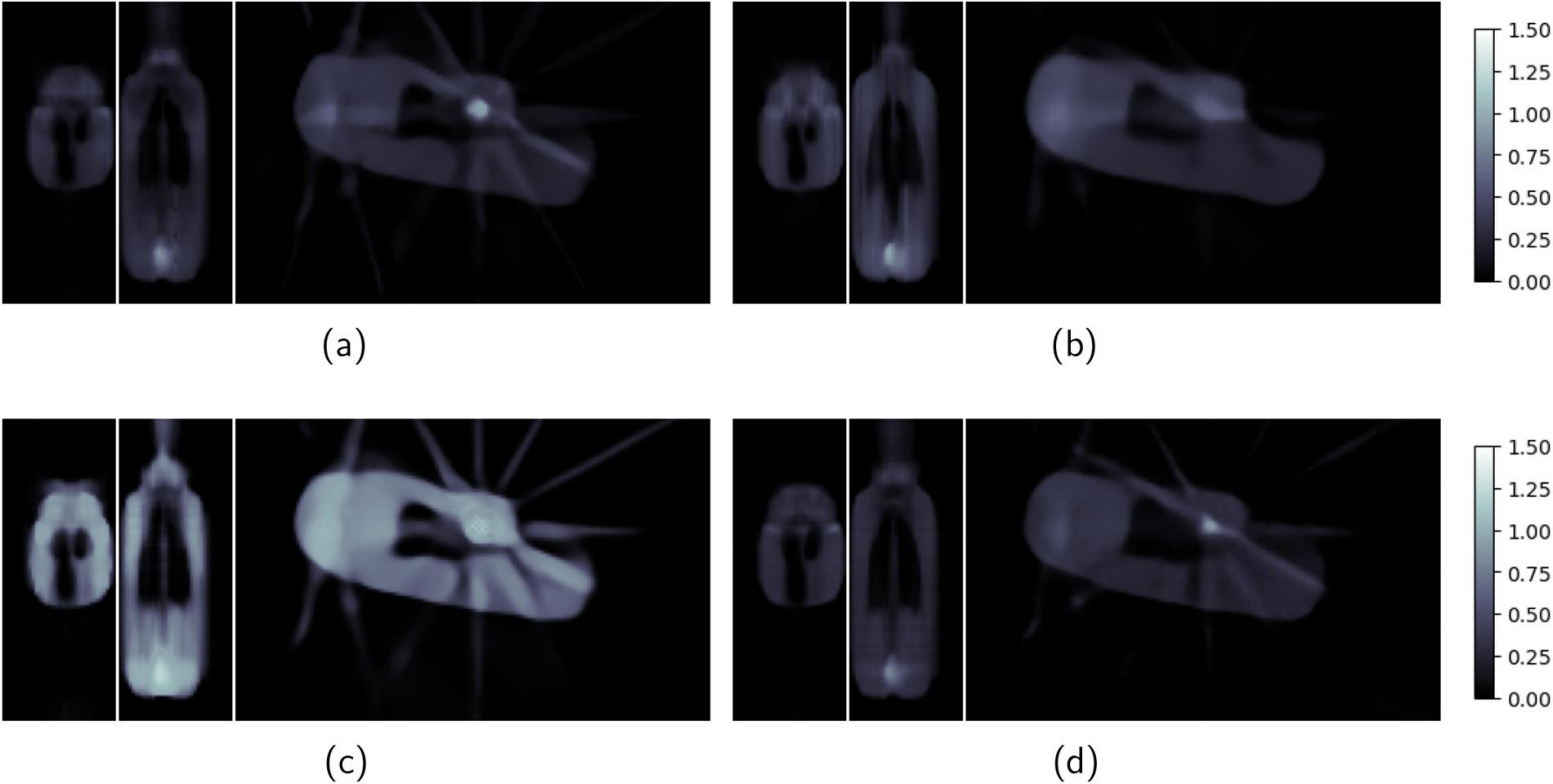
Comparison of mouse-like phantom reconstructions using various proposed transformer-based LPD algorithms and the UNet-based LPD algorithm. **(a)** UNet-based LPD. **(b)** Stacked Transformer-based LPD. **(c)** Restormer-based LPD. **(d)** UNet-based LPD+CAB.

Figures 12, 13 present the reconstructions of the experimental data using the MLEM algorithm and all LPD variants. Compared to the synthetic test results, these reconstructions exhibit greater variability. The UNet-based LPD produces a relatively distorted image with beam-like artifacts originating from the activity sources. The UNet-based LPD+CAB shows a slight improvement over the UNet-based LPD but remains inferior to the Stacked Transformer-based LPD. The Dual-Domain Stacked Transformer-based LPD yields a more homogeneous reconstruction, although some residual artifacts remain. The Dual-Domain Restormer-based LPD generates a brighter image but with more pronounced beam-like artifacts.

From a qualitative perspective, reconstructions with uniform intensity within each organ and across the body are preferred, since the underlying activity distribution in these regions is expected to be uniform. Based on this criterion, the Dual-Domain Stacked Transformer-based LPD provides the most consistent reconstruction. In contrast, the UNet-based LPD variants (both UNet-based LPD and UNet-based LPD+CAB) exhibit higher local contrast in hot regions but at the cost of increased artifacts.

It should be noted that in this study, “uniformity” refers to the visual consistency of reconstructed intensity values within homogeneous regions of the image. It is assessed qualitatively through visual inspection by comparing reconstructed outputs across methods. No numerical uniformity index is computed.

## 4 Qualitative evaluation

### 4.1 Visualization of the unrolling

**Figure 14:**
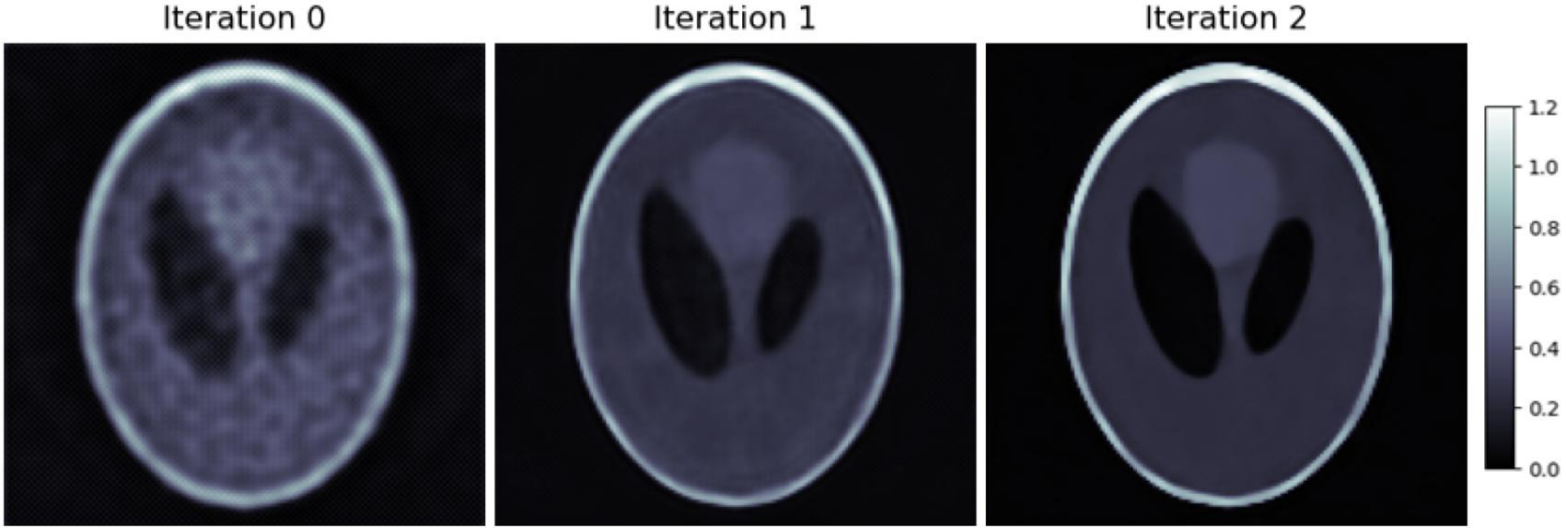
Reconstruction outputs across different iterations of the U-Net-based LPD algorithm.

**Figure 15:**
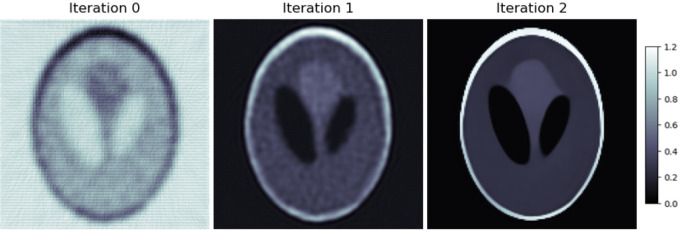
Reconstruction outputs across different iterations of the Restormer-based LPD algorithm.

**Figure 16:**
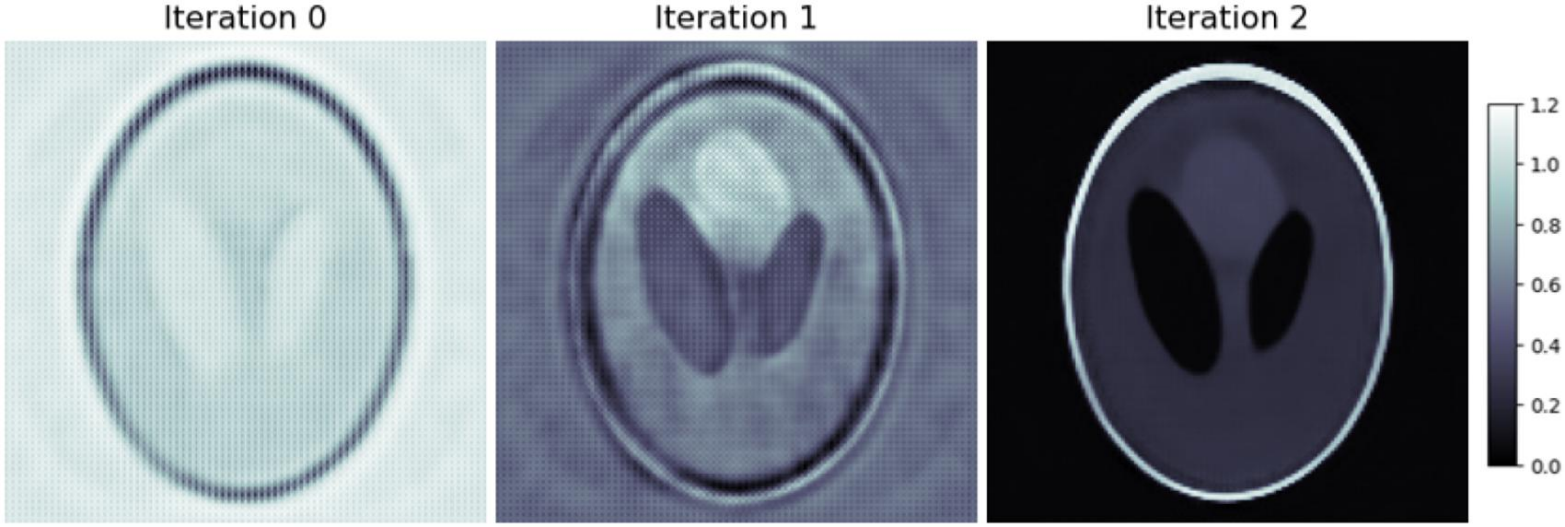
Reconstruction outputs across different iterations of the Stacked Transformer-based LPD algorithm.

**Figure 17:**
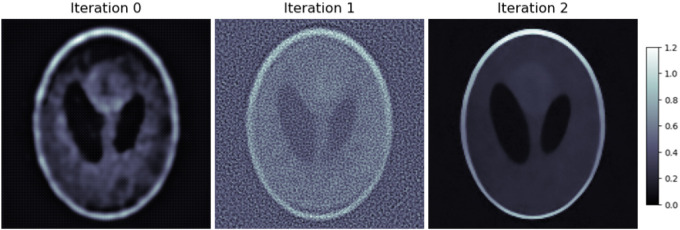
Reconstruction outputs across different iterations of the UNet-based LPD+CAB algorithm.

To better understand how each model operates across iterations, the outputs of intermediate primal-channel iterations are visualized in Figures 14–17. These visualizations illustrate how different architectures progressively refine the reconstruction process.

For the UNet-based LPD (Figure 14), the reconstruction process demonstrates a more gradual and stable refinement behavior across iterations. The first iteration yields a nonuniform estimate of the phantom structure with relatively high contrast. In the second iteration, structural boundaries become more distinguishable, and global anatomical features are better recovered. The final iteration further enhances edge definition and contrast, although the level of fine structural detail remains less pronounced compared to transformer-based variants. Overall, the UNet-based LPD shows consistent iterative improvement, but with comparatively limited capacity for capturing fine-grained details.

For the Dual-Domain Restormer-based LPD, the intermediate results (Figure 15) show a clear progression from a relatively over-smoothed representation and globally stabilized estimate in the first iteration toward increasingly refined structures in later iterations. This behavior suggests that the model initially suppresses noise and reconstructs a coarse global structure, followed by iterative enhancement of anatomical details and boundary sharpness.

In contrast, the Stacked Transformer-based LPD (Figure 16) exhibits a different reconstruction trajectory. The first iteration provides a strongly smoothed representation of the phantom, while later iterations introduce more pronounced structural components, including improved separation of inner regions. However, residual artifacts and texture inconsistencies remain visible in intermediate stages, indicating a less stable refinement path compared to the Restormer-based model. The final iteration produces a sharper reconstruction, but with some localized intensity fluctuations.

For the UNet-based LPD incorporating cross-attention (Figure 17), the intermediate reconstructions remain relatively noisy with limited recovery of fine structural details compared to the standard UNet-based LPD. However, the final reconstruction shows clearer improvements, outperforming the baseline in terms of visual quality and structural detail preservation. These results suggest that the CAB module further enhances the refinement process, indicating that cross-attention can alleviate the inherent locality limitations of the CNN-based backbone.

### 4.2 Model robustness

To evaluate reconstruction robustness, we compute the pixel-wise variance of reconstructed images obtained from multiple noise realizations of the Shepp–Logan phantom at a noise level of 0.3, as described in Section 2.4.2.

**Figure 18:**
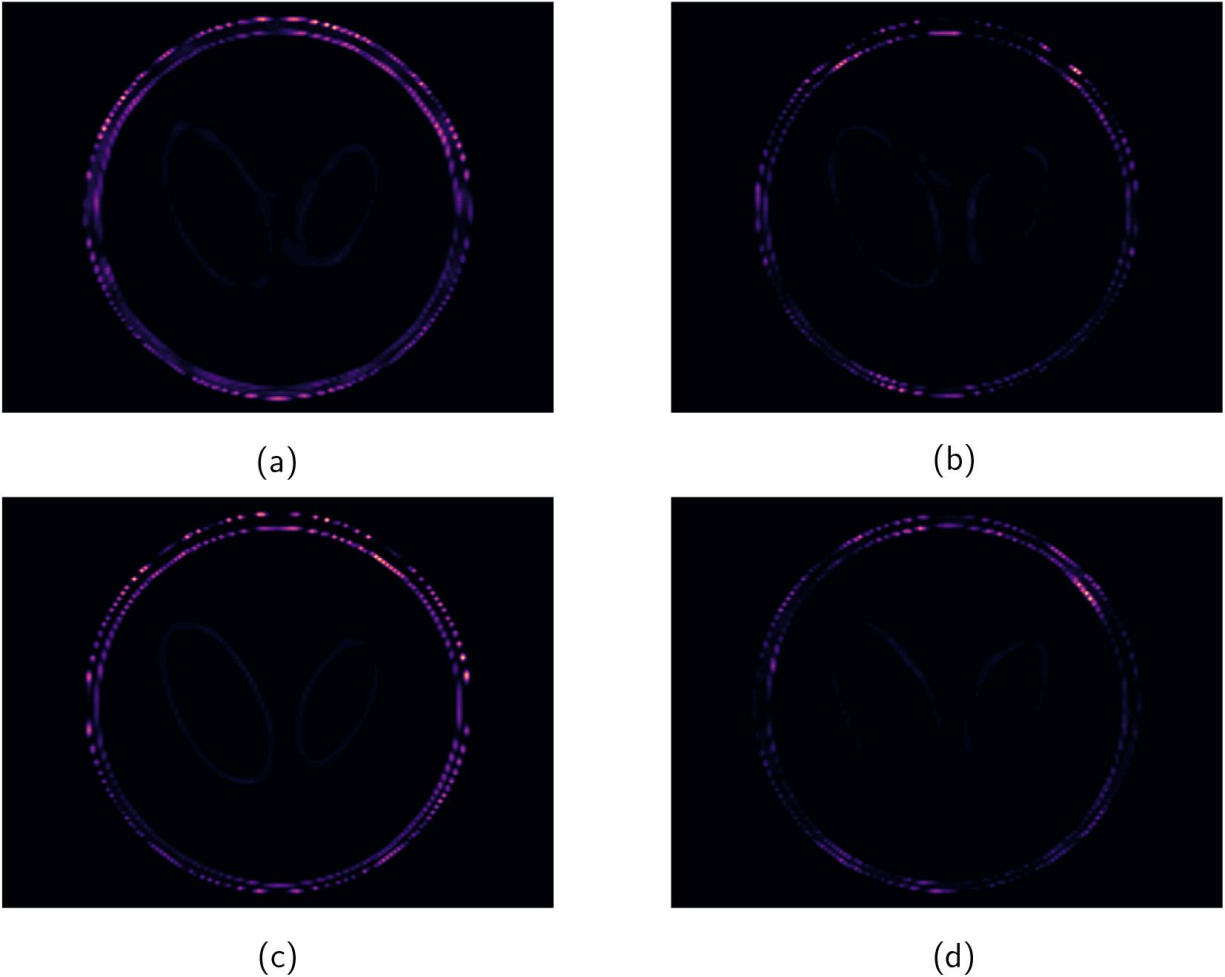
Variance of the model outputs at a noise level of 0.3. **(a)** UNet-based LPD. **(b)** Stacked Transformer-based LPD. **(c)** Restormer-based LPD. **(d)** UNet-based LPD+CAB.

As shown in Figure 18, this analysis highlights the regions most sensitive to noise. The Dual-Domain Restormer-based LPD demonstrates reduced variance in certain areas of the shell, whereas the UNet-based LPD shows comparatively higher variance around the cavities. While higher noise sensitivity is generally expected in regions with greater activity, this factor alone does not fully explain the elevated variance near the cavities or the reduced variance observed in the central parts of the shell.

## 5 Discussion

In this section, we analyze and interpret the experimental results obtained from both synthetic and experimental datasets, with a particular focus on the behavior of different LPD architectures under varying data conditions. These observations are consistent with recent Transformer-based CT and PET reconstruction studies, where global attention mechanisms have been shown to improve structural preservation compared to CNN-based designs, particularly in low-dose or low-count settings.

Overall, the quantitative results demonstrate that all proposed LPD variants consistently outperform the UNet-based baseline in terms of MSE and PSNR across all noise levels. Among them, the Restormer-based LPD achieves the best performance at low noise levels, while the UNet-based LPD with cross-attention (CAB) provides the most favorable results at moderate to high noise levels. The Stacked Transformer-based LPD also improves intensity-based metrics, although to a lesser extent compared to the other proposed models. In terms of SSIM, the improvements are less pronounced, with some methods exhibiting slightly lower values than the baseline. This behavior suggests a trade-off between pixel-wise accuracy and structural similarity, where gains in MSE and PSNR do not always translate into improved structural preservation. Overall, these findings indicate that the Restormer-based LPD offers strong performance under low-noise conditions, while the CAB-enhanced UNet provides more robust behavior as noise increases, achieving a favorable balance between reconstruction accuracy and structural fidelity. This behavior can be attributed to the integration of cross-attention within the LPD framework, which enhances feature interactions beyond the locality constraints of CNNs. In contrast, the Stacked Transformer-based LPD, while effective in improving intensity reconstruction, shows comparatively weaker preservation of fine structural details.

Interestingly, the ranking observed on synthetic data does not hold on experimental data, where the Stacked Transformer-based LPD provides the most consistent and visually plausible reconstructions. While the Restormer-based LPD produces brighter images, it also introduces more pronounced beam-like artifacts, suggesting increased sensitivity to real-world inconsistencies such as noise mismatch, scatter, attenuation inaccuracies, and other system modeling errors. In contrast, the Stacked Transformer-based LPD achieves a better balance between global context modeling and robustness, leading to more homogeneous activity distributions across organs. The UNet-based LPD produces distorted reconstructions with noticeable beam-like artifacts, reflecting its inability to capture long-range dependencies in the sinogram domain. The UNet-based LPD with cross-attention also exhibits higher local contrast in hot regions but at the cost of relatively increased artifacts. These findings provide insight into the motivation of this work. While transformer-based architectures help alleviate the locality limitations of CNN-based sinogram processing, the extent of this benefit depends on the specific architecture. Global and cross-attention mechanisms can enhance structural consistency and reduce artifacts; however, stronger global modeling does not necessarily translate into improved experimental performance, likely due to increased sensitivity to domain shift. This highlights a trade-off between representational power and robustness.

The discrepancy between synthetic and experimental performance can be attributed to differences in data realism. Synthetic data rely on accurate forward models and simplified noise assumptions, whereas experimental data include complex noise and physical effects not fully captured during training. These factors can introduce biases in learned representations and affect generalization to real measurements, particularly under domain shift conditions.

To further investigate whether the observed improvements of the proposed architectures are primarily attributed to increased model capacity, we evaluated an expanded UNet-based LPD baseline with four unrolled iterations (UNet-based LPD (4 Iterations)). Increasing the number of iterations improved the performance of the UNet-based LPD, demonstrating that additional capacity contributes to reconstruction quality. However, the performance improvements were not solely determined by the increased number of parameters. The UNet-based LPD (4 Iterations) remained inferior to the Restormer-based LPD and UNet-based LPD+CAB models across the evaluated metrics, while showing competitive performance compared with the Stacked Transformer-based LPD. These results suggest that both model capacity and architectural design influence reconstruction performance. In particular, the ability of attention-based modules to capture long-range dependencies and cross-domain interactions contributes to the improved reconstruction quality beyond the effect of parameter count alone.

It is important to note that the experimental evaluation in this study is limited to a single phantom, providing only preliminary evidence for the generalization of models trained purely on synthetic data to real measurements. Furthermore, the lack of anatomical annotations and a voxel-wise reference activity distribution limits quantitative region-based evaluation. Despite this limitation, the results offer an important initial validation on real preclinical data, demonstrating the feasibility of transferring the proposed approach beyond the synthetic domain. Future work will focus on extending the evaluation to a broader range of both synthetic and experimental PET datasets to more comprehensively assess robustness and generalization under diverse acquisition conditions. In addition, future improvements may include iteration-aware attention mechanisms to better leverage the progressive refinement process in LPD reconstruction. Extending the attention design to capture spatio-temporal dependencies within the LPD framework may represent another promising direction.

## 6 Conclusions

This study explored the potential of Dual-Domain Transformer-based LPD algorithms for PET image reconstruction, aiming to merge the interpretability of model-based iterative methods with the representational power of deep learning. To this end, we proposed and evaluated three novel transformer-based LPD architectures: the Dual-Domain Stacked Transformer-based LPD, the Dual-Domain Restormer-based LPD, and the UNet-based LPD incorporating Cross-Attention Blocks (CABs). These architectures leverage attention mechanisms to capture global contextual relationships in both the sinogram and image domains, while preserving the iterative refinement benefits of unrolled reconstruction. On synthetic data, all proposed LPD variants consistently outperform the UNet baseline in MSE and PSNR across all noise levels. The Restormer-based LPD performs best under low noise, while the CAB-enhanced UNet is more robust at moderate to high noise levels. The Stacked Transformer also improves intensity metrics but to a lesser extent. SSIM improvements are less consistent, indicating a trade-off between pixel-wise accuracy and structural preservation. Overall, Restormer performs best in low-noise conditions, whereas CAB provides better robustness at higher noise levels via cross-attention, while the Stacked Transformer shows weaker structural detail preservation. On experimental data, the ranking changes, with the Stacked Transformer-based LPD providing the most stable and homogeneous reconstructions. The Restormer-based LPD shows increased beam-like artifacts, likely due to higher sensitivity to real-world data inconsistencies and domain shift, while UNet-based LPD exhibits more pronounced degradation. The UNet-based LPD with cross-attention increases local contrast in hot regions, while also introducing a slight increase in artifacts. Overall, the results indicate that attention mechanisms enhance global context modeling, but their effectiveness is architecture-dependent. The proposed synthetic data generation strategy, tailored to the MiniPET-3 geometry with Gaussian blurring and Poisson noise, enables effective training, but the observed synthetic-to-experimental gap highlights missing physical effects in the simulation. In conclusion, transformer-based LPD architectures offer a promising direction for low-count PET reconstruction, with a trade-off between representational power and robustness under real-world conditions.

## Data Availability

The raw data supporting the conclusions of this article will be made available by the authors, without undue reservation.
